# Psychosocial Impact of Multiple Sclerosis on Couples: Relationship Between Anxiety, Depression, and Stress Communication of Both Partners

**DOI:** 10.1177/21501319221119142

**Published:** 2022-08-30

**Authors:** Ada-Katrin Busch, André Fringer

**Affiliations:** 1 Faculty of Health, Department of Nursing Science, Witten/Herdecke University, Witten, Germany; 2 School of Health Science, Institute of Nursing, Zurich University of Applied Sciences, Winterthur, Switzerland

**Keywords:** couples, multiple sclerosis, anxiety, depression, communication, development of intervention

## Abstract

**Background:**

Multiple Sclerosis (MS) influences the relationships of affected couples, whereby the disease-related stress can lead to a deterioration of communication. This, in turn, makes it difficult for the couples to cope successfully. To support couples affected by MS for coping with the disease, the first step in developing an intervention is to examine whether this situation also applies in the Swiss context.

**Methods:**

A cross-sectional study was conducted to examine the psychosocial situation of couples where 1 partner has MS, regarding anxiety, depression, and stress communication. The Hospital Anxiety and Depression Scales (HADS-D) were used to assess depression and anxiety in both partners of 462 couples, while their stress communication was assessed using questions formulated according to the corresponding subscales of the Dyadic Coping Inventory (DCI). A comparison of the assessments of both partners was performed using the Mann-Whitney *U* test. Furthermore, the relationship between their stress communication and the severity of anxiety and depression was calculated using Spearman’s rank correlation.

**Results:**

Life partners rated the stress communication of their partners with MS significantly higher than the partners with MS themselves. Moreover, life partners could not distinguish whether their partners with MS expressed a sense of burden or a need for support. These findings indicate that the stress communication skills of both partners show potential for optimization. Health status regarding depression and anxiety revealed the following: 34.2% of the persons with MS and 34% of their life partners experienced clinically high levels of anxiety (HADS-D/A ≥ 8.0), and 31.4% of those with MS and 20.2% of the life partners showed clinically high levels of depression (HADS-D/D ≥ 8.0).

**Conclusion:**

In the Swiss context, psychosocial intervention, which includes communication training for both partners, might be effective in improving the health status regarding depression and anxiety as well as the stress communication.

## Introduction

Multiple sclerosis (MS) is a chronic, progressive, and often disabling autoimmune disease, characterized by inflammation, demyelination, and neurodegeneration of the central nervous system. Approximately 2.5 million people worldwide are affected, with women dominating men in a ratio of 2:1 to 3:1.^1,2^ In Switzerland, there are approximately 15 000 people living with MS.^3^ There is still no cure for MS, but long-term disease-modifying therapies to slow its progression and alleviate symptoms of relapsing-remitting MS do exist.^4^

The course of the disease is difficult to predict due to its heterogeneity, and the fact that various symptoms occur depending on the size and location of the inflammation. For both partners in a couple, this uncertainty can trigger psychosocial problems such as anxiety and depression, thereby reducing their quality of life.^5^ Thus, it is not surprising that anxiety and depression are highly prevalent in persons with MS.^6-8^ Earlier research has ascertained that both, persons with MS and their life partners, report a strong fear or a feeling of panic caused by the uncertain future or a possible worsening of the disease.^9^ Over half of the people with MS (40%-60%) experience one or more depressive episodes during the trajectory of their disease.^10-12^ By comparison, such disorders occur in 20% of the general population.

Consequently, MS changes the lives of both the partners. Necessary changes in professional, family and relationship roles can, at times, be experienced as extremely intense and threatening.

Though until recent decades, studies only examined the individual perspectives of people with MS and their partners without MS, they have now begun to study couples living with MS. While some of these studies focus on the experience of illness, and examine the interaction processes in the relationship as well as the underlying interaction and negotiation processes of coping,^13,14^ others look at the burden of disease, as well as the dyadic appraisal, coping, and adjustment.^15-20^

It has been shown that couples who are convinced that they can manage life with a chronic illness together are those who succeed in supporting each other in their adjustment process.^21,22^ Therefore, it is particularly important for both partners to talk openly about the changing physical and psychological stress factors like anxiety, despair, and exhaustion. It is these shared conversations that allow partners to adjust to coping together.^23^ However, excessive stress quickly leads to a deterioration in communication.^24^

To develop an intervention that could strengthen MS-affected couples in coping with the disease, in congruence with the MRC framework,^25^ we evaluated the initial situation for the Swiss context. This is done by screening for the presence of depression and anxiety in both partners of MS-affected couples and determining the extent to which they communicate their stress or burden and need for support in the target population.

Therefore, the present study is guided by the following research questions:

1. Do both partners where one is affected by MS suffer from anxiety and depression? If so, to what extent?
2. How often do the partners communicate their distress and support needs?
3. For each the person with and without MS, is there an individual relationship between the frequency of communicating their distress or support needs, and the severity of their anxiety and depression?
4. For partners with MS, is there a relationship between their self-assessment and their life partner’s (external) assessment of their stress communication?

## Methods

### Design and Participants

This cross-sectional survey was part of a research study,^26^ approved by the Ethics Committee of Basel, Switzerland and conducted on the situation and the needs of people with MS as well as those of one family member. In this study, 2700 persons with MS were enrolled. Inclusion criteria were a diagnosed MS and the ability to complete a questionnaire in German. The relative was a person close to the affected person, not necessarily the life partner.

Of the 878 people with MS (32%) who responded to the survey, 615 (70.1%) of the relatives participated in the study mentioned. The analysis of the study at hand is performed on the datasets derived from the 462 pairs formed by persons with MS and their partners (hereafter referred to as life partners).

### Data Collection and Analysis

Sociodemographic data as well as disease-related data were collected. To analyze these datasets, the frequency of each answer was counted, and a percentage was determined for every data analysis category. To screen for the presence and severity of anxiety and depression in both partners, the questionnaire included the “Hospital Anxiety Depression Scale” (HADS-D) in its full German version.^27^ The total sum of the point values per subscale was then assigned to its appropriate diagnostic group “unremarkable” (≤7), “borderline” (8-10), and “conspicuous” (≥11).

**Figure 1.**
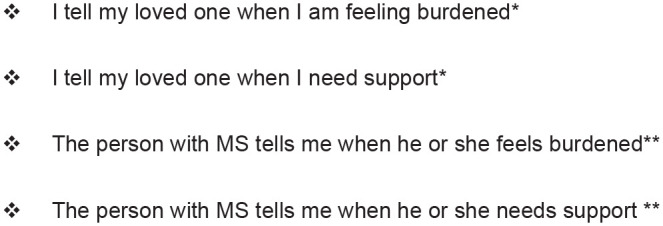
Items on self-assessment and external assessment of stress expression. *Included in the questionnaire of the person with MS as well as in the questionnaire of the life partner. **Included in the questionnaire of the life partner.

To collect information on how often both partners communicate about their perceived burden and their needs of support, the items shown in Figure 1 were formulated based on the stress expression subscales of the Dyadic Coping Inventory (DCI).^28^

These items were answered using a five-point Likert scale where zero represented “very rare” and 5 represented “very often.” To evaluate the stress communication, the absolute and relative frequencies of every answer were counted. Because external assessment by the life partners was also present in the stress communication data, it was possible to identify a match/mismatch between the self-assessment and external assessment. To verify the significance of possible differences, the Mann-Whitney *U* test with a significance level of .05 was computed. The relationship between the stress communication and the severity of the anxiety and depression was computed using Spearman’s rank correlation, where the correlation was significant at the level of .05 (2-sided *P* > .05). The Statistical Package for Social Sciences (SPSS Version 28.0) was used for data analysis.

## Results

The statistical analysis in the present study was based on a dataset of 462 couples consisting of persons with MS and their life partners. Of the 462 couples, in more than half (55.6%), the female partner had MS. The course of their disease was unknown to 26 (5.6%) of the 450 persons with MS who answered this item. 157 (34%) of the individuals reported that their disease was diagnosed as relapsing remitting MS (RRMS). In 149 (32.3%), the disease progressed to secondary progressive MS, and in 118 (25.5%) individuals, the disease showed a progressive course from onset (PPMS). While the partners with MS were between 20 and 88 years old (*M* = 54.00, SD = 12.31), the age range of their life partners was between 17 and 84 years (*M* = 54.27, SD = 12.37). Partners affected by MS have been diagnosed with MS between less than 1 to 58 years (*M* =15.67, SD = 10.90), while the life partners indicated that they have been providing support for a minimum of less than a year to a maximum of 58 years (*M* = 11.51, SD = 9.26).

Furthermore, the disease Course of people with MS and their functional limitations are presented in Tables 1 and 2, while anxiety and depression status of the whole sample (people with MS and their life partners) can be found in Table 3.

**Table 1.** Disease Courses of Partners With MS.

	Partners with MS (N462)
Relapsing remitting (RRMS)	34.0% (n = 157)
Primary progressive (PPMS)	25.5% (n = 118)
Secondary progressive (SPMS)	32.3% (n = 149)
Unknown	5.6% (n = 26)
Missing	2.6% (n = 12)

**Table 2.** Functional Limitations in Partners With MS.

	Partners with MS (N462)
Not/slightly	12.8% (n = 59)
Few/effort is hard	14.7% (n = 68)
No employment possible, many impairments but rarely need assistance	37.7% (n = 174)
Depend on regular assistance	16.5% (n = 76)
Independence severely limited, most of the time in wheelchair or bed	16.0% (n = 74)
Missing	2.4% (n = 11)

**Table 3.** Prevalence of Anxiety and Depression in Partners With MS and Life Partners.

	Partners with MS (N462)	Life partners (N462)
Anxiety		
Unremarkable (HADS-D/A ≤ 7)	65.8% (n = 304)	66% (n = 305)
Borderline (HADS-D/A 8-10)	20.3% (n = 94)	17.5% (n = 81)
Conspicuous (HADS-D/A ≥ 11)	13.9% (n = 64)	16.5% (n = 76)
Depression		
Unremarkable (HADS-D/D ≤ 7)	68.6% (n = 317)	79.9% (n = 369)
Borderline (HADS-D/D 8-10)	18.2% (n = 84)	11.6% (n = 54)
Conspicuous (HADS-D/D ≥ 11)	13.2% (n = 61)	08.6% (n = 39)

**Figure 2.**
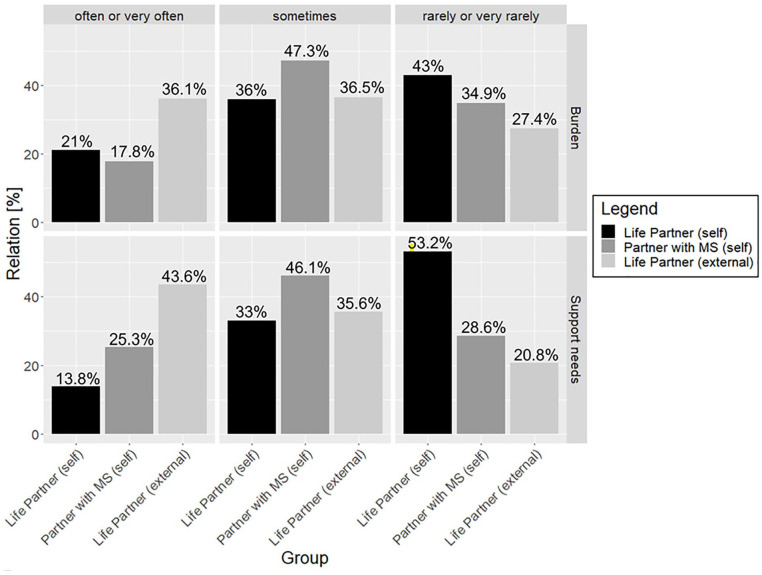
Self- and external assessments of stress expression.

Regarding the stress communication, 461 of the 462 couples answered 2 questions, assessing the frequency with which they expressed their sense of burden (Figure 2).

The differences between the assessments of persons with MS and their life partners were not significant (*P* = .74).

Regarding communicating the need for support (Figure 2), there was a significant difference (*P* < .01) between the assessments of persons with MS and their life partners.

Concerning the questions of stress communication, in 457 of the 462 couples, a self-assessment by the partner with MS and an external assessment by the life partner (referred in Figure 2 as “Partner with MS (external)”) exists. Significant differences were found in both, in the comparison of the assessments regarding the frequency of expressions of burden (*P* < .01) and the frequency of expressions of support needs (*P* < .01).

### Correlations Between Stress Communication, Anxiety, and Depression

#### Person with MS

The more often the partners with MS communicated their sense of burden, the lower the severity of their depression symptoms (*r_s_* = −.132, *P* = .004, n = 461) were (Table 4). Partners with MS who exhibited higher levels of anxiety were less likely to communicate the need for support than those with MS who exhibited lower levels of anxiety (*r*_s_ = −.177, *P* = .000, n = 460).

**Table 4.** Correlations Between Stress Communication, Anxiety, and Depression.

	My partner (with MS) tells me openly that they experience stress or burden	My partner (with MS) tells me openly that they would appreciate my support	Anxiety in life partners (HADS-A in sum)	Depression in life partners (HADS-D in sum)	Anxiety in partners with MS (HADS-A in sum)	Depress-ion in partners with MS (HADS-D in sum)
The partner with MS lets the life partner know, that they experience stress or burden						
Correlation coefficient	.307**	.214**	.053	.004	−.007	−.132**
Significance (2 tailed)	.000	.000	.256	.940	.880	.004
N	458	459	460	460	461	461
The partner with MS lets the life partner know that they appreciate support						
Correlation coefficient	.196**	.369**	−.009	−.026	−.177**	−.067
Significance (2 tailed)	.000	.000	.854	.576	.000	.152
N	457	458	459	459	460	460
The life partner lets the partner with MS know, that they experience stress or burden						
Correlation coefficient	.298**	.199**	.071	.005	−.077	−.020
Significance (2 tailed)	.000	.000	.129	.922	.101	.676
N	460	461	461	461	459	459
The life partner lets the partner with MS know that they appreciate support						
Correlation coefficient	.181**	.208**	.090	−.008	−.120*	−.063
Significance (2 tailed)	.000	.000	.055	.866	.010	.180
N	460	461	461	461	459	459
My partner (with MS) tells me openly that they experience stress or burden						
Correlation coefficient	1000	.607**	−.065	−.091	.103*	−.029
Significance (2 tailed)	.	.000	.164	.050	.028	.531
N	460	460	460	460	459	460
My partner (with MS) tells me openly that they would appreciate my support						
Correlation coefficient	.607**	1000	.064	.072	−.020	.121**
Significance (2 tailed)	.000		.167	.125	.670	.009
N	460	461	461	461	459	460

* Correlation is significant at the .05 level (2-tailed).

** Correlation is significant at the .01 level (2-tailed).

#### Life partners

For life partners, there were no significant correlations between their stress communication and the severity of their anxiety and depression (Table 4). However, the more often the life partners perceived that their partners with MS expressed burden, the more often they also perceived that their partners with MS expressed their need for support (*r_s_* = .607, *P* = .000, n = 460).

#### Self-assessment of partners with MS and external assessment by life partners

Persons with MS with life partners who more frequently hear their partner with MS communicate a sense of burden, experience a higher degree of anxiety (*r_s_* = .103, *P* = .028, n = 459) than partners with MS with life partners who less often recognize that the partner with MS expresses their sense of burden (Table 4). Persons with MS with life partners who more frequently hear that the partner with MS communicates their need of support experience a higher degree of depression (*r_s_* = .121, *P* = .009, n = 460) than persons with MS with life partners who less often recognize that the partner with MS expresses their need of support.

### Differences in Correlations Between Self and External Assessments

Correlations performed further point out the stress communication between both partners: the more the partners with MS communicate their stress, the more frequent the life partners notice that the partner with MS expresses stress, or vice versa (Table 4). This could be the sense of burden (*r_s_* = .307, *P* = .000, n = 458) or need of support (*r_s_* = .214, *P* = .000, n = 459). If the partner with MS communicates their need of support, the life partner notices this with similar frequency (*r_s_* = .196, *P* = .000, n = 457), however, they also notice that the partner with MS has expressed their sense of burden (*r_s_* = .369, *P* = .000, n = 458). Furthermore, the more often the partner without MS communicates feelings of burden, the more they perceive that the partner with MS also expresses a sense of burden (*r_s_* = .298, *P* = .000, n = 460) or the need for support (*r_s_* = .199, *P* = .000, n = 461). If the life partner often expresses a need for support, they notice more frequently, that the partner with MS has communicated their feelings of burden (*r_s_* = .181, *P* = .000, n = 460) along with the need for support (*r_s_* = .206, *P* = .000, n = 461).

## Discussion

The purpose of this study was to improve knowledge about whether and to what extent couples living with MS in Switzerland are affected by depression and anxiety, and how often they express their sense of burden as well as their need for support.

In accordance with the literature,^7,9,10^ an increased prevalence on a specific cut-off date and increased lifetime prevalence of anxiety and depression in persons with MS and their life partners, support the findings of this study, which indicate high levels of anxiety (HADS-D/A ≥ 8.0) and depression (HADS-D/D ≥ 8.0) in both partners.

The results of the present study show that 31.4% of partners with MS and 20.2% of their life partners suffered from depression. Other authors confirm these findings.^9,29,30^ A possible reason for this difference is that the mental health of the partners with MS may also be threatened by organic brain changes and medication.^31^

Regarding the level of anxiety, we found that 34% of the life partners had clinically relevant levels of anxiety (score ≥8.0), compared with 34.2% of partners with MS. Similar results were obtained by Janssens et al^17^ In their longitudinal study, 40% of the life partners and 34% of recently diagnosed partners with MS suffered from high levels of anxiety. During their 2-year follow-up, according to the authors, the high levels of anxiety remained unchanged in partners with MS and their life partners, leading the authors to hypothesize that the high levels of anxiety continue in the long term. Since in our study group, partners with MS have been living with the disease for an average of 15.67 years (SD = 10.90) and their cohabiting partners have been supporting them for an average of 11.51 years (SD = 9.26), we agree with this hypothesis.

According to the respective self-assessments, persons with MS express a need for support more often than their partners. A possible reason for this could be disease-related limitations, but consistent with the relationship-based coping approach, this could also be a sign of dysfunctional support behavior of the life partners (eg, protective buffering/overprotection).^32^

The Systemic Transactional Model posits that adequate communication of stress, and the response of the partner, are important for stress regulation process at the individual and the dyadic level.^33^ The supportive life partner without MS must perceive and decode the partner`s signs of stress, which is facilitated by a clear expression of stress.^21^ The fact that in our study, life partners rated the stress communication of the partners with MS significantly higher than the partners with MS themselves, as well as the circumstance that the life partners perceived the stress communication of the partners with MS but were not able to differentiate between their partner`s expressions of burden and their expressions of support needs, led us to the conclusion that there is no in-depth understanding about the communication of stress. Additionally, the present study showed that the communication about stress of life partners correlated positively with that of partners with MS. This result could also indicate that clear messages were not sent, and the respective responses did not meet the needs of the speaker. However, to be able to react appropriately to the communication, a clear expression is necessary. If this is not successful, misunderstandings, conflicts and emotional distance quickly arise and lead to incongruent perceptions of common dyadic coping along with higher psychological distress.^34^

According to the GFB guideline,^35^ care becomes necessary when current or impending health problems interfere with the independent performance of daily activities and require companionship in the experience of illness or support from family members.

The results of this study state the presence of depression and anxiety, as well as dysfunctional communication patterns in both partners with MS and their life partners.

Based on these findings, it is hypothesized that nursing interventions to improve the stress communication within couples can support partners’ individual well-being by reducing the levels of depression and anxiety as well as improving the overall satisfaction with the partnership.

### Limitations

Because this is a cross-sectional study, no conclusions can be drawn about the causality. Additionally, this study does not claim to fully explain the phenomena. Rather it is intended to verify that the increased incidence of more severe depression and anxiety, and the frequency with which couples living with a chronic illness communicate, described in the international literature for MS patients and their life partners, applies to the corresponding target group in Switzerland. Therefore, confounding factors, such as gender and length of time since diagnosis, may have influenced the results.

## Conclusion

In conclusion, couples living with MS experience high levels of anxiety and depression. While persons with MS communicate their stress slightly more often than life partners, a significant correlation does exist between the frequencies in which partners with MS communicate their stress and the severity of their anxiety and depression. A more frequently used stress communication and the ability to properly perceive and decode the expressions of burden and the need for support within the couples lead to a better control of the stressors, and a decrease in the feelings of anxiety and depression. Based on these findings, there is a need for clinical practice to improve stress communication within couples, as this is likely to optimize couples’ psychosocial situations in terms of depression and anxiety, as well as their partnership satisfaction.
